# Reply to Lauro, A.; Ripoli, M.C. Comment on “Klek et al. Enhanced Recovery after Surgery (ERAS) Protocol Is a Safe and Effective Approach in Patients with Gastrointestinal Fistulas Undergoing Reconstruction: Results from a Prospective Study. *Nutrients* 2021, *13*, 1953”

**DOI:** 10.3390/nu14010018

**Published:** 2021-12-22

**Authors:** Stanislaw Klek

**Affiliations:** Surgical Oncology Clinic, The Maria Sklodowska-Curie National Cancer Institute, 31-115 Krakow, Poland; klek@poczta.onet.pl

We would like to thank you for the opportunity to respond to the issues raised in Dr Lauro’s and Ropoli’s letter, to clarify aspects of our methodology in relation to these concerns. We would also like to thank both Doctors for their interest in our paper and for taking the time to express their concerns.

In their letter to the editor, Dr Lauro and Ropoli said that the surgery for gastrointestinal (GI) fistula is one of the most challenging and prone to complications surgical procedures, and they were absolutely right. These are demanding operations. Nonetheless, we can improve the outcome by implementing a particular strategy, which is based on team work and prehabilitation. Firstly, however, I wanted to confirm our results, as follows: 0% mortality rate for both groups and 34.4 vs. 14.5% complication rates [1]. Secondly, I wanted to clarify the following surgical approach: these were final repairs, not emergency procedures. Thirdly, I have to explain how we work with our patients. Our surgical unit is the part of Intestinal Failure Center at the Stanley Dudrick’s Memorial Hospital in Skawina, Poland, which is, among other activities, also the center for home parenteral nutrition (HPN). We have recently published a summary of the last twenty years of our activity [2]. Being the HPN center gives us unique and comfortable chance to prepare our patients for the final surgery over months, sometimes years, always under our surveillance. The multidisciplinary team (MDT) cover all patient needs. Nutrition is a key component of prehabilitation, and the other aspects of the latter comprise all Enhanced Recovery After Surgery (ERAS) Protocol’s principles. That is how our patients become fit for surgery.

One more time, on behalf of all Authors I wanted to express my gratitude to the Editor and our Italian Colleagues.
